# “*Candidatus* Gortzia shahrazadis”, a Novel Endosymbiont of *Paramecium multimicronucleatum* and a Revision of the Biogeographical Distribution of *Holospora*-Like Bacteria

**DOI:** 10.3389/fmicb.2016.01704

**Published:** 2016-11-04

**Authors:** Valentina Serra, Sergei I. Fokin, Michele Castelli, Charan K. Basuri, Venkatamahesh Nitla, Franco Verni, Bhagavatula V. Sandeep, Chaganti Kalavati, Giulio Petroni

**Affiliations:** ^1^Department of Biology, University of PisaPisa, Italy; ^2^Department of Invertebrate Zoology, Saint Petersburg State UniversitySaint Petersburg, Russia; ^3^Department of Veterinary Medicine, University of MilanMilan, Italy; ^4^Department of Zoology, Andhra UniversityVisakhapatnam, India; ^5^Department of Biotechnology, Andhra UniversityVisakhapatnam, India

**Keywords:** Gortzia, *Holospora*, *bacteria*, *Paramecium*, endosymbiosis, distribution, India

## Abstract

*Holospora* spp. and “*Candidatus* Gortzia infectiva”, known as *Holospora*-like bacteria (HLB), are commonly found as nuclear endosymbionts of ciliates, especially the *Paramecium* genus. HLB are related by phylogenetic relationships, morphological features, and life-cycles, which involve two alternating morphotypes: reproductive and infectious forms (RF, IF). In this paper we describe a novel species belonging to the “*Ca*. Gortzia” genus, detected in *P. multimicronucleatum*, a ciliate for which infection by an HLB has not been reported, discovered in India. This novel endosymbiont shows unusual and surprising features with respect to other HLB, such as large variations in IF morphology and the occasional ability to reproduce in the host cytoplasm. We propose the name of “*Candidatus* Gortzia shahrazadis” for this novel HLB. Moreover, we report two additional species of HLB from Indian *Paramecium* populations: “*Ca*. Gortzia infectiva” (from *P. jenningsi*), and *H. obtusa* (from *P. caudatum*); the latter is the first record of *Holospora* from a tropical country. Although tropical, we retrieved *H. obtusa* at an elevation of 706 m corresponding to a moderate climate not unlike conditions where *Holospora* are normally found, suggesting the genus *Holospora* does exist in tropical countries, but restricted to higher elevations.

## Introduction

Ciliates (Alveolata, Ciliophora) have long been known to be potential hosts for bacteria (Preer et al., 1974; Görtz, 1983; Heckmann and Schmidt, 1987; reviewed in Görtz, 1996, 2006; Fokin, 2004a, 2012; Fujishima, 2009a). Molecular characterization shows a large biodiversity among these symbionts (Irbis and Ushida, 2004; Vannini et al., 2004, 2010, 2014; Rinke et al., 2006; Schrallhammer et al., 2006, 2011, 2013; Ferrantini et al., 2009; Boscaro et al., 2012, 2013a,b,c; Gong et al., 2014; Senra et al., 2015; Szokoli et al., 2016) and also the type of interaction with their hosts can range from mutualistic to parasitic (Kusch et al., 2002; Vannini et al., 2003, 2007; Fels and Kaltz, 2006; Fellous et al., 2011). In this work, we use De Bary's definition of “symbiosys” (de Bary, 1879), as “the living together of two differently named organisms”, independent of effects on the organisms involved, thereby including mutualism, parasitism, and commensalism. The terms “symbionts” and “endosymbionts” also use this definition.

*Holospora* is a well-known genus of endosymbiotic bacteria. The *Holospora* name, meaning “whole spore” was invoked by Hafkine (1890), to describe rod-shaped bacteria, similar to spores, infecting a French population of *P. caudatum* (found in an aquarium), including *H. obtusa, H. undulata* and *H. elegans* (Hafkine, 1890; redescribed in Gromov and Ossipov, 1981; Preer and Preer, 1982). Since then, many other *Holospora* species have been described (although most lack a proper description according to the bacterial taxonomic code), such as “*H. acuminata*”, “*H. bacillata*”, *H. caryophila*, “*H. curvata*”, “*H. curviuscula*”, “*H. recta*”, and *Holospora* spp. (Ossipov et al., 1980; Preer and Preer, 1982; Borchsenius et al., 1983; Fokin, 1991; Fokin and Sabaneyeva, 1993; Fokin et al., 1999, 2006; reviewed in Görtz and Schmidt, 2005; Ferrantini et al., 2007). The *Holospora*-like bacteria (HLB) group includes all known *Holospora* species and “*Candidatus* Gortzia infectiva”, recently found in a stable endosymbiosis in the *P. jenningsi* macronucleus (MA) (Boscaro et al., 2013a). These two genera of bacteria share similarities in morphology, in life-cycles and show a close phylogenetic relation (Boscaro et al., 2013a).

*Holospora*-like bacteria (HLB) are Gram-negative, non-motile *Alphaproteobacteria*, inhabiting either the MA or the micronucleus (MI) of *Paramecium* species (for review see Görtz and Schmidt, 2005; Fokin and Görtz, 2009), as well as the MA of *Frontonia* (Fokin et al., 2006; Ferrantini et al., 2007).

All HLB live in an obligate endosymbiosis with their hosts, and have variable degrees of nuclear and host specificity (Ossipov, 1973; Fujishima and Görtz, 1983; Görtz, 1983; Fokin, 2000; Boscaro et al., 2013a; for review on *Holospora* see Fujishima, 2009b). They undergo a distinctive life-cycle involving two different forms: the smaller and almost round reproductive form (1–3 μm) (RF), and the much more elongated, rod-like infectious form (4–20 μm) (IF), in which it is possible to recognize several cell parts: cytoplasm, periplasm, and an apical structure, the “recognition tip” (Görtz and Diekmann, 1980; Görtz et al., 1989, 1990; Fujishima et al., 1990; Boscaro et al., 2013a).

*Holospora*-like bacteria (HLB) can be spread by both horizontal and vertical transmission (reviewed in Fokin and Görtz, 2009). The IFs invade a new host cell via a phagocytotic pathway and, after acidosome fusion, are able to escape from digestive vacuoles and reach the nucleus of the ciliate (Görtz and Wiemann, 1989) via movement mediated by the host cytoskeleton (Fokin et al., 2003; Sabaneyeva et al., 2005, 2009). Inside the nucleus, IFs differentiate into reproductive forms, which divide by binary fission. During host cell division, RFs are shared between two daughter nuclei. In most *Holospora* species (i.e., “*H. acuminata*”, “*H. curviuscula*”, *H. elegans, H. obtusa*, “*H. recta*”, and *H. undulata*) the majority of infectious forms are concentrated in a central part of the dividing nucleus—called the “connecting piece”—that will be released into the environment (Wiemann and Görtz, 1989). The remaining *Holospora* species (i.e., “*H. bacillata*”, *H. caryophila*, “*H. curvata*”) and “*Ca*. Gortzia infectiva” do not show this particular localization (Fokin et al., 1996; Boscaro et al., 2013a; Fokin, 2015), and instead are released from the host by an inverted path of infection (Fokin and Sabaneyeva, 1997; Fokin, 2015): IFs are released singularly or in small groups from the nucleus into the cytoplasm, and later into the environment.

*Holospora* species have been widely recorded by protistologists, allowing researchers to produce a biogeography of these endosymbionts (Fokin and Görtz, 2009), although the infection rate in *Paramecium* populations is not always permanent and follows cyclic fluctuations (Fokin and Görtz, 2009; Duncan et al., 2015).

Although the genus *Paramecium* is nearly cosmopolitan (Fokin, 1997, 2010/11; Przyboś and Fokin, 2000), *Holospora* have only been isolated in cold to temperate areas, in the north of America, Asia and Europe (Hori and Fujishima, 2003; Fokin, 2004b; Fokin et al., 2006). However, this pattern is most likely biased by the lack of sampling in tropical countries, where sampling efforts have been limited (Fokin et al., 2004; Görtz, 2008; Fokin and Sera, 2014). An exception is “*Ca*. Gortzia infectiva”, which has only been found in a sample from Thailand, (Boscaro et al., 2013a), showing that HLB can exist in tropical climates.

In this paper, we report the retrieval of HLB from another tropical area, the south of India. We found and described a novel species of HLB, which is the first reported from *P. multimicronucleatum*. Moreover, we found “*Ca*. Gortzia infectiva” in the MA of an Indian population of *P. jenningsi* and, quite surprisingly, also *H. obtusa* infecting the MA of *P. caudatum* from India. Our records represent the first report of “*Ca*. Gortzia infectiva” from India and the first finding of the *Holospora* genus in a tropical country (although from a higher elevation with a moderate climate), at the lowest latitude ever reported. Thus, our study provides new and important information about HLB distribution, reshaping HLB biogeography.

## Materials and methods

### *Paramecium* collection and identification

Sampling was carried out in India, during 2014: *P*. *multimicronucleatum* population TP2 was collected in freshwater Kolleru Lake, Andhra Pradesh (N 16°44′16.0″ E 81°24′18.0″; 28th September); *P. multimicronucleatum* PC6 strain was sampled in Pedda Cheruvu, the largest freshwater water body in the Vizianagaram district, Andhra Pradesh (N 18°06′36.0″ E 83°24′0.0″; 23rd November); *P. jenningsi* population BJ1 was collected in the brackish water Chilka Lake, Odisha (N 19°44′37.0″ E 85°12′44.4″; 3rd February); *P. caudatum* population SH2 was collected in a freshwater stream on Araku Hills, 706 m above the sea level, in Visakhapatnam district, Andhra Pradesh (N 18°16′32.3″ E 83°02′15.6″; 27th January). Where not indicated, samples were collected at sea level. The Araku hills have a moderate climate: the average maximum day-time temperature is 32.6°C and the average minimum temperature is 24.0°C. During the Indian summer season (March-June) the average maximum temperature is 35.2°C, the minimum is 26.2°C (http://www.yr.no/place/india/Andhra_Pradesh/Araku/statistics.html).

Samples were grown at room temperature and fed Cerophyll medium inoculated with *Raoultella planticola* (see Boscaro et al., 2013a for details). Unfortunately the SH2 and BJ1 populations survived only a few weeks in laboratory conditions, but for *P. multimicronucleatum* population TP2 we were able to create a monoclonal culture, named TP2-2.

Living observations and images were made with an Axio Lab.A1 (Zeiss) microscope and/or by an Orthoplan Leitz microscope equipped with differential interference contrast (DIC), as well as a Leica DMR microscope at × 300–1250 magnifications. 18S rDNA sequencing was performed to confirm morphological identifications. About 20 cells for each population were isolated and stored in 70% Ethanol at −20°C until genomic extraction using a NucleoSpin™ Plant II kit (Macherey-Nagel, Germany). The polymerase chain reaction (PCR) was carried out with the following primers: 18S F9 (5′- CTG GTT GAT CCT GCC AG -3′), (Medlin et al., 1988), and 18S R1513Hypo (5′- TGA TCC TTC YGC AGG TTC -3′), (Petroni et al., 2002). All PCRs were performed in a 40 μl reaction volume with 0.25 μl primers (100 μM), TaKaRa PCR reagents and ExTaq (Takara Bio, Japan) using a C1000™ Thermal Cycler (Bio-Rad, Hercules, CA). The PCR program used was: denaturation at 94°C for 30 s, annealing at 55°C for 30 s, elongation at 72°C for 2 min, and final elongation step at 72° for 6 min. PCR products were cleaned with the EuroGOLD Cycle-Pure kit (EuroClone, Milano, Italy) and sent to GATC Biotech Company (Germany) for sequencing with the following internal primers: 18S R536 (5′-CTG GAA TTA CCG CGG CTG-3′), 18S R1052 (5′-AAC TAA GAA CGG CCA TGC A-3′) and 18S F783 (5′-GAC GAT CAG ATA CCG TC-3′), (Rosati et al., 2004).

### Characterization of endosymbionts

Examination of living cells show that SH2, BJ1, and TP2/TP2-2 paramecia exhibit MA bacterial infections. Transmission electron microscopy (TEM) analysis was performed on the TP2-2 monoclonal strain following the protocol in Boscaro et al. (2013a).

We carried out cross-infection experiments using infected TP2-2 cells as donor, and the endosymbiont-free *P. multimicronucleatum* strain PC6 as recipient. Experimental infection was carried out by preparing a homogenate of infected cells according to Preer (1969). Recipient cells were infected by mixing equal volumes of cell culture with the donor cell homogenate in a 3 mL depression slide, and incubating at room temperature. In order to check the infection status a set of living cells (*n* = 10) was observed by DIC after 2, 24 and 48 h after mixture with the homogenate. We could test the trans-infection with only one species of *Paramecium* as receiver, due to the small number of cells in the TP2 culture.

Endosymbiont 16S sequences were obtained for all the populations using the *Alphaproteobacteria* universal primers 16S Alfa F19b, 16S R1522b (Table 1); sequencing was initially carried out using *bacterial* universal primers (16S F343 ND, 16S F785 ND, 16S R515 ND); subsequently we designed specific sequencing primers for each species (for details see Table 1). For the TP2 endosymbiont, two additional PCR reactions were performed with two different sets of primers: 16S Alfa F19b together with 16S Gortz R659 (annealing temperature was changed to 51°C) and 16S F114HoloCaedi together with 16S R1488 Holo. The first PCR product was sequenced using R534 Gortzia, the second with F1008 Gortzia and 16S R1328HoloCaedi, and the sequencing results assembled into a single sequence. The 16S rRNA gene sequences were aligned using the ARB software package (Ludwig et al., 2004) and manually checked against more than 600,000 bacterial sequences from SSU rRNA SILVA 123 Ref NR 99 database (Quast et al., 2013). For phylogenetic analyses 58 sequences were employed: together with the 3 new sequences, 48 selected sequences belonging to order *Rickettsiales* and 7 sequences belonging to class *Alphaproteobacteria* as outgroup (sequences not shown in the tree are listed in Table S1). The alignment was reduced in length, producing a 1632 character matrix. Maximum likelihood (ML) analyses (PHYML 5.3.2) (Guindon and Gascuel, 2003) and Bayesian inference (BI) analyses (MrBayes 3.2) (Ronquist et al., 2012) were performed, with the GTR +I +G substitution model, as indicated by AIC (Akaike's information criterion), calculated by jModelTest 2.2 (Darriba et al., 2012). ML analysis was applied with 1000 pseudoreplicates, while for BI analysis, three different Markov Chain Monte Carlo runs were employed, with one cold chain and three heated chains each, running for 500,000 generations.

**Table 1:** **List of primers used for 16S rRNA encoding gene sequencing of *Holospora*-like bacteria from *Paramecium* spp**.

**Name**	**Sequence (5′–3′)**	**Target**	**Use**	**Type**	**Specificity**	**References**
16S Alfa F19b^a^	CCTGGCTCAGAGCGAACG	GS, GI, HO	PCR, sPCR	Forward	Most *Alphaproteobacteria*	Modified from Vannini et al., 2004
16S R1522b	GGAGGTGATCCAACCGCA	GI, HO	PCR	Reverse	Most *Alphaproteobacteria*	Schrallhammer et al., 2006
16S Gortz R659^a^	TTCCGTTTTCCTCTACCA	GS	sPCR	Reverse	Genus “*Ca*. Gortzia”	Adapted from Boscaro et al., 2013a
16S F114HoloCaedi^b^	TGAGTAACGCGTGGGAATC	GS	sPCR	Forward	Some *Rickettsiales*	Boscaro et al., 2013a
16S R1488 Holo^b^	TACCTTGTTACGACTTAACC	GS	sPCR	Reverse	Some *Rickettsiales*	Boscaro et al., 2013a
16S F343 ND	TACGGGAGGCAGCAG	GS, HO	SEQ	Forward	Most *Bacteria*	Vannini et al., 2004
16S F785 ND	GGATTAGATACCCTGGTA	GS, HO	SEQ	Forward	Most *Bacteria*	Vannini et al., 2004
16S R515 ND	ACCGCGGCTGCTGGCAC	GS, HO	SEQ	Reverse	Most *Bacteria*	Vannini et al., 2004
R418 Holo_obt	GGGCTTTTTCTCTCGTTACC	HO	SEQ	Reverse	*Holospora obtusa*	Present work
F881 Holo	TTACCGCGGCGGCTGGCA	HO	SEQ	Forward	Genus *Holospora*	Present work
R1143 Holo	GAACTTTTTCTCTCGCTACC	HO	SEQ	Reverse	Genus *Holospora*	Present work
16S R1328HoloCaedi	TAGCGATTCCAACTTCATG	GS, GI	SEQ	Reverse	Some *Rickettsiales*	Boscaro et al., 2013a
R534 Gortzia	CACGCTTTCGTGCCTCA	GS, GI	SEQ	Reverse	Genus “*Ca*. Gortzia”	Present work
F1008 Gortzia	AGCTCTTTTACTCGTGAAG	GS, GI	SEQ	Forward	Genus “*Ca*. Gortzia”	Present work

GS, “Candidatus Gortzia shahrazadis” (TP2); GI, “Ca. Gortzia infectiva” (BJ1); HO, Holospora obtusa (SH2); sPCR selective PCR

a Primer used in selective PCR to obtain the initial segment of 16S rDNA

b *Primer used in selective PCR to obtain the final segment of 16S rDNA*.

Fluorescence *In Situ* Hybridization (FISH) analyses was performed as described by Boscaro et al. (2013a). A first set of experiments was carried out with *P. jenningsi* BJ1 and *P. multimicronucleatum* TP2 using a probe designed for “*Ca*. Gortzia infectiva”, GortProb659 (5′-TTCCGTTTTCCTCTACCA-3′), (Boscaro et al., 2013a), labeled with a cyanine 3 (Cy3) fluorophore at the 5′ end, together with a *Bacterial* universal probe EUB338 (word5′-GCTGCCTCCCGTAGGAGT-3′) (Amann et al., 1990), labeled with fluorescein isothiocyanate (FITC) at the 5′ end. We then designed two new species-specific probes able to distinguish between the two endosymbionts from *P. jenningsi* and *P. multimicronucleatum*, since GortProb659 was found to label both. We designed GortzInf_1268 (word5′-TCCTGATTCGCTCAAGGTC-3′; FITC fluorophore in 5′ end), specific for “*Ca*. G. infectiva”, and GortzSha_1266 (word5′-TTTTGATTTGCTCAAGGTCGC-3′; Cy3 fluorophore in 5′ end), specific for the new HLB from *P. multimicronucleatum*. Both probes were tested *in silico* on the RDP (ribosomal database project) (Cole et al., 2009) and SILVA database using TestProbe 3.0 (Quast et al., 2013), allowing 0 mismatches. It was not possible to experimentally test GortzInf_1268 because *P. jenningsi* BJ1 infected with “*Ca*. Gortzia infectiva” was lost. We tested GortzSha_1266 (Cy3) in competition with GortzInf_1268 (FITC) or EUB338 (FITC) on the *P. multimicronucleatum* endosymbiont.

## Results

### *Paramecium* species identification

**Figure 1:**
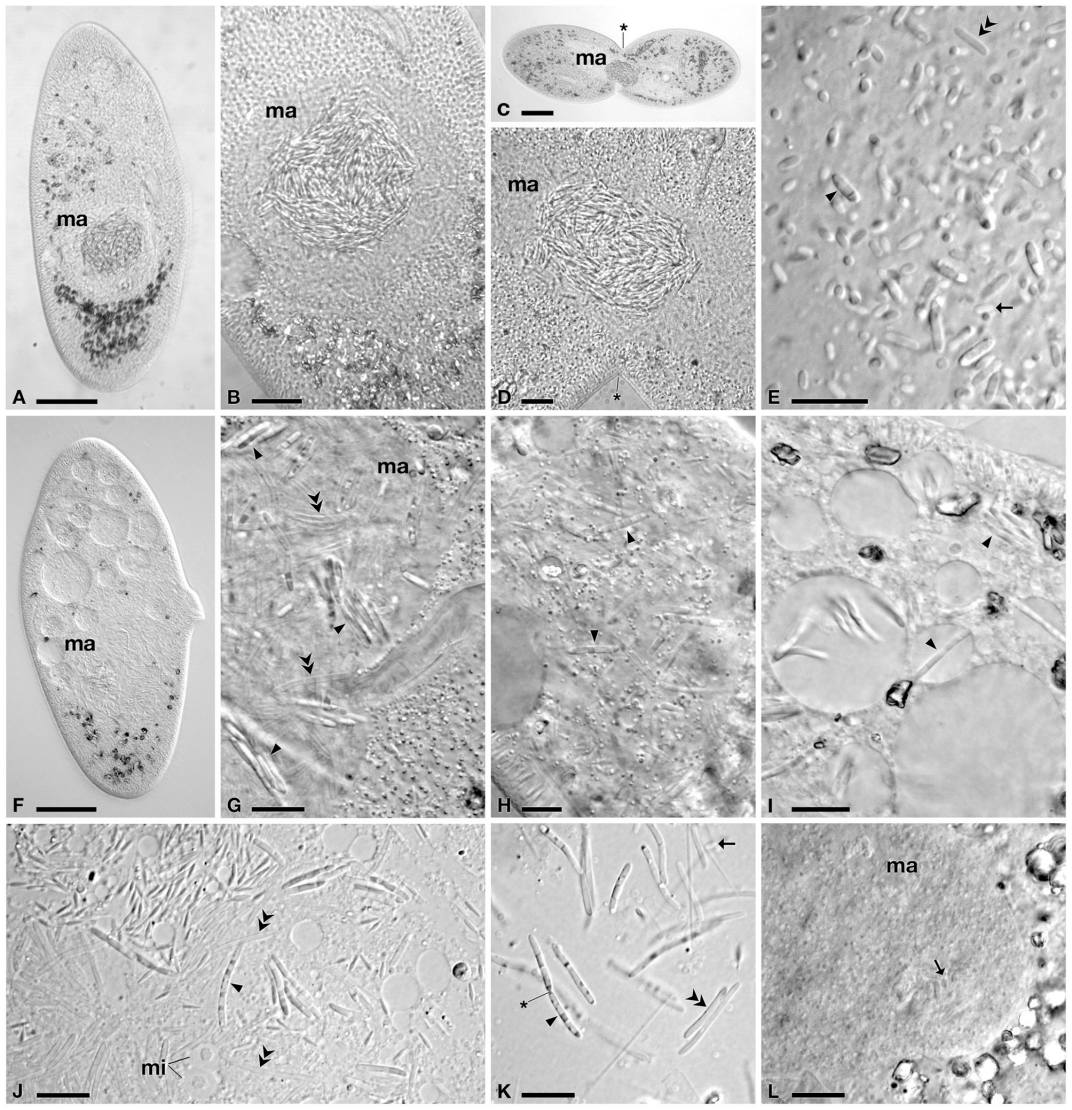
**Light microscopy of infected cells of *P. multimicronucleatum*. (A–E)** TP2 cells a few weeks after environmental sampling and **(F–K)** after 1 year in culture. **(A)** Whole cell with macronuclear infection. **(B)** Infected macronucleus (*ma*), with bacteria clustering inside. **(C)** Infected cell undergoing division and **(D)** detail of its *ma*, showing absence of connecting piece. **(E)** Bacterial forms released after *ma* squashing, showing classical size and morphology of reproductive (*rf*) and infectious (*if*) forms. **(F)** Whole cell with macronuclear infection after 1 year of cultivation, in which bacterial cells are less visible. **(G)** Detail of infected *ma*, in which *if* cluster together in small groups, among a large number of transient forms (*tf*). **(H)** *if* in host cytoplasm. **(I)** Detail of *if* in host cytoplasm inside and outside vacuoles. **(J)** Detail of host vesicular type micronuclei (*mi*) and *if*, showing some changes in morphology, such as size and unusual striated/dotted pattern. **(K)** *if* undergoing binary fission and *tf* after cell squashing. **(L)** *ma* of strain PC6, infected by bacteria during cross-infection experiments, showing classical *rf* morphology. *Arrowheads* indicate infectious forms, *arrows* reproductive forms and *double arrowheads* transient forms. The *asterisk* indicates the cleavage furrow. *Bars* stand for 30 μm **(A,F)**, 20 μm **(C,H)** and 10 μm **(B,D,E,G,I–L)**.

**Figure 2:**
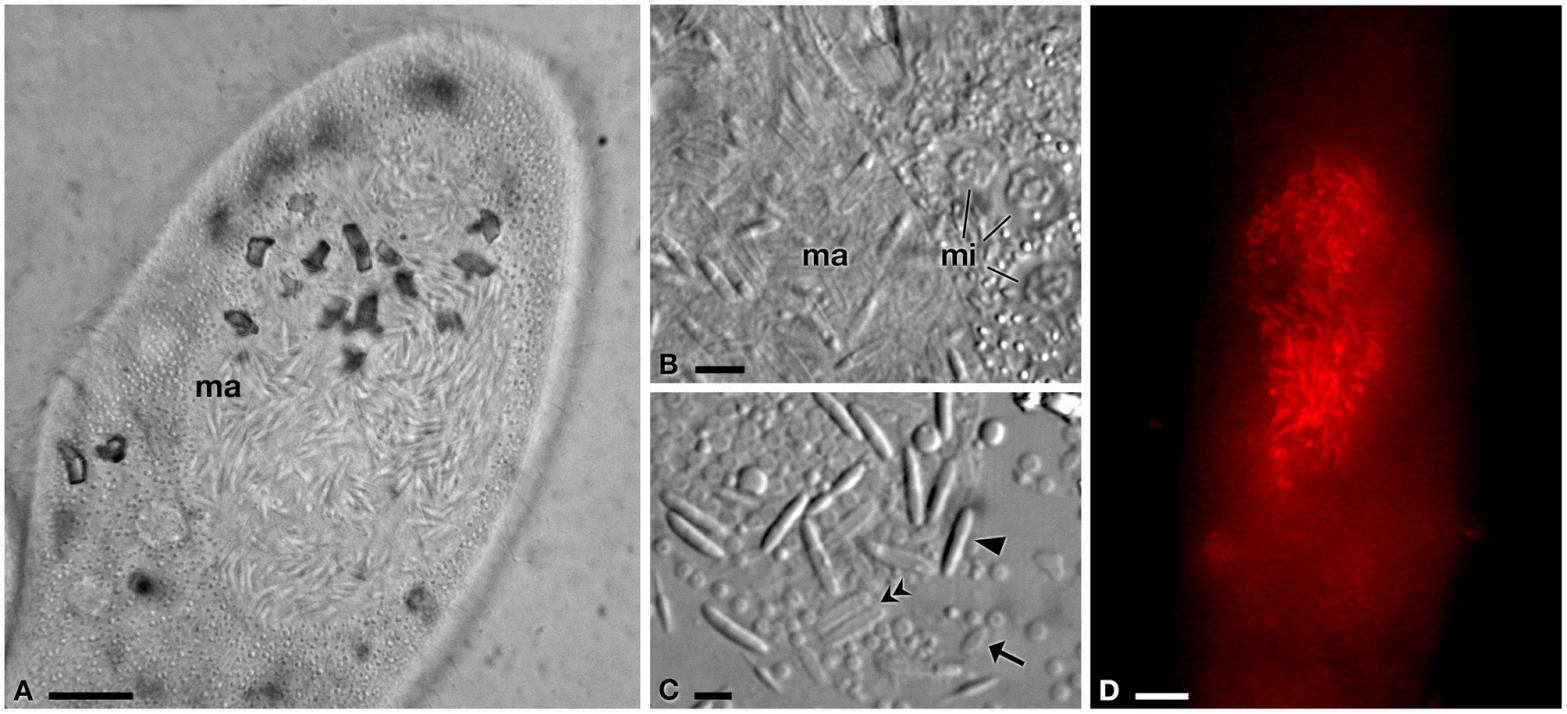
**Light microscopy and FISH analyses of infected *P. jenningsi* BJ1. (A)** Cell with hyperinfected macronucleus (*ma*). **(B)** Infected *ma* and three chromosomal type micronuclei (*mi*). **(C)** Detail of reproductive, infectious and transient forms, after *ma* crushing. **(D)** Positive signal of the probe GortProb659 (labeled with Cy3, emitting in red) inside host *ma*, during FISH experiment. *Arrowheads* indicate infectious forms, *arrows* reproductive forms and *double arrowheads* transient forms. *Bars* stand for 10 μm **(A,D)** and 5 μm **(B,C)**.

**Figure 3:**
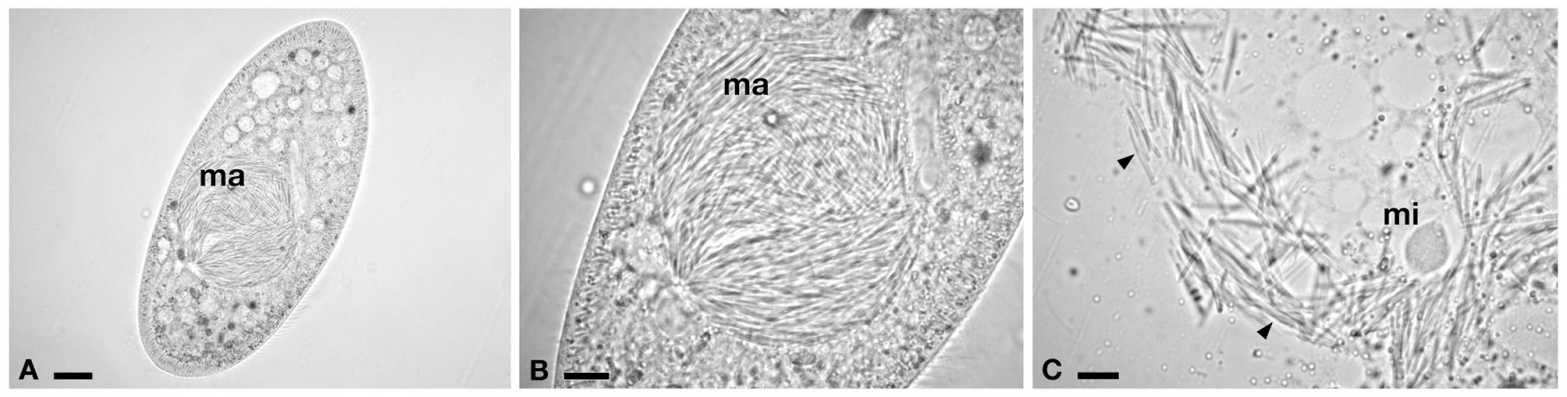
**Light microscopy of infected *P. caudatum* SH2. (A)** Whole cell with macronuclear infection. **(B)** Detail of hyperinfected macronucleus (*ma*). **(C)** Compact type micronucleus (*mi*) and rod-shaped bacteria, released after *ma* squashing. *Arrowheads* indicate infectious forms. *Bars* stand for 20 μm **(A)** and 10 μm **(B,C)**.

*Paramecium* TP2 and PC6 exhibited the typical cell morphology and size of *P. multimicronucleatum* morphospecies (Figure 1) (Fokin, 1997, 2010/11; Fokin and Chivilev, 2000), showing the presence of several vesicular-type MI (Figure 1J). We obtained two identical 1710 bp 18S rDNA sequences that matched more than 10 *P. multimicronucleatum* sequences (including HG315606, HE662762, and other) with 99.35–99.88% identity. For this reason, we identify our TP2 and PC6 organisms as *P. multimicronucleatum*. Nevertheless, our sequences reached only 98.49% identity if compared to another group of *P. multimicronucleatum* sequences, including the one for the morphospecies (AF255361) (Strüder-Kypke et al., 2000). Such variability in 18S rDNA sequences inside the taxon suggests the presence of cryptic species, a situation that will be addressed in forthcoming studies. *Paramecium* BJ1 was identified as *P. jenningsi* (Figure 2), due to the presence of two chromosomal-type MI (strangely, in some cases, more than two) (Fokin, 1997) (Figure 2B) and by 18S rDNA sequence (1711 bp) identity with *P. jenningsi* (HE662760, AF100311) of 99.88%. We identified *Paramecium* SH2 as *P. caudatum* (Figure 3) because it exhibited one compact-type MI (Figure 3C) and its 18S rDNA sequence (1710 bp) matched *P. caudatum* HE664171 with 99.00% identity, with 1 indel and 16 mismatches. This number of mismatches is unusual in 18S rDNA sequences of the same *Paramecium* species, suggesting again a possible cryptic *P. caudatum* species. These newly obtained sequences are available from the ENA database: LT549005 (*P. multimicronucleatum* TP2); LT549006 (*P. multimicronucleatum* PC6); LT549003 (*P. jenningsi* BJ1); LT549004 (*P. caudatum* SH2).

### Characterization of new HLB from *P. multimicronucleatum* TP2

Cells of *P. multimicronucleatum* TP2 exhibited 100% MA infection by rod shaped bacteria (Figure 1). A few weeks after isolation *Paramecium* cells manifested hyperinfected MAs, with dense clusters of bacteria (Figures 1A,B). The symbionts showed the typical morphology of HLB, with two different forms: the small RF (2.5–3.3 μm) and the larger, rod shaped IF, (6.9–10.7 μm) (Figure 1E). The IF were characterized by slightly tapered ends and by darker/reflecting parts in their bodies. In addition, some transient forms (TF)—large but not containing reflecting material—were present (Figure 1E). One year after isolation and subcloning, we repeated the analysis to check the infection status of TP2-2 and noticed that the distribution of the bacteria in the MA had changed: IF were clustered together in several small groups inside the nucleus (Figures 1F,G). IF and TF increased their length in comparison to previous observations, reaching 8.2–14.7 μm and 6.7–15.5 μm, respectively. In addition, the number of TF increased dramatically, whereas classical RF were fewer and/or less visible, compared to the first observations. We also observed IF dividing by binary fission (Figure 1K). Moreover, many IF showed an abnormal distribution of periplasmic parts inside the cell, forming a striated/dotted pattern (Figures 1J,K).

**Figure 4:**
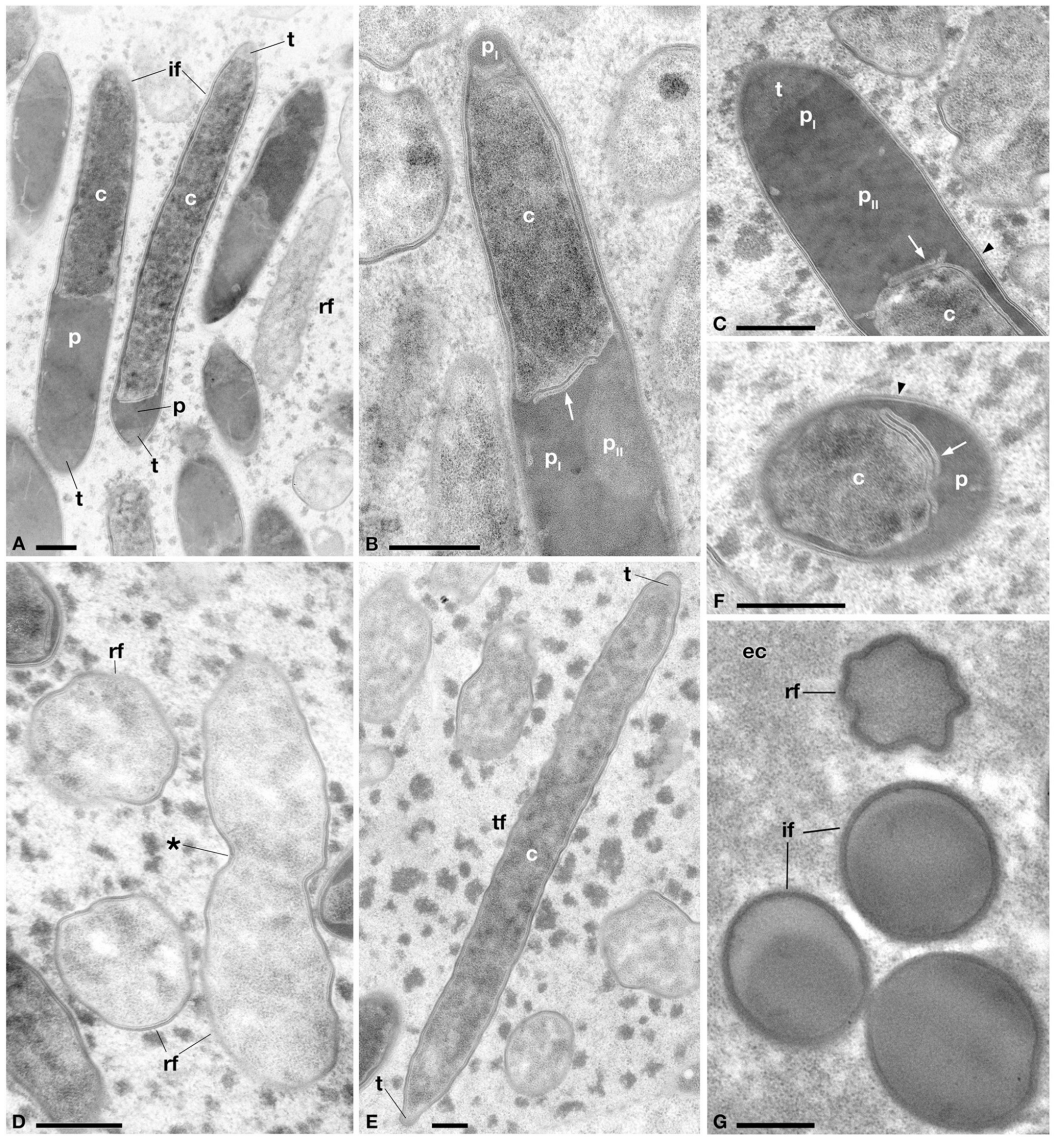
**Ultrastructural morphology of the novel *Holospora*-like bacteria harbored by *P*. *multimicronucleatum*, after 1 year of cultivation. (A)** Longitudinal section of reproductive (*rf*) and infectious (*if*) forms, the latter showing various degrees of cell compartmentalization in cytoplasm (*c*), periplasm (*p*) and recognition tip (*t*). **(B)** Detail of *if* showing cytoplasmic extrusion and periplasm with two electron-dense areas, a darker (*p*_*I*_) and a lighter (*p*_*II*_) one. **(C)** Detail of *t, p*_*I*_, and *p*_*II*_ in *if*. **(D)** *rf* in transverse section and in longitudinal section during binary fission. **(E)** Transient form (*tf*) in longitudinal section with *t* at both ends of cellular body. **(F)** Transverse section of *if* showing irregular distribution of *p* around cytoplasmic part. **(G)** Transverse section of *if* and *rf* inside eukaryotic cytoplasm (*ec*) of host. *Arrows* indicate cytoplasmic extrusion in periplasmic space, *arrowhead* indicates the membrane layers surrounding IF. The *asterisk* indicates the cleavage furrow. *Bars* stand for 0.5 μm.

**Figure 5:**
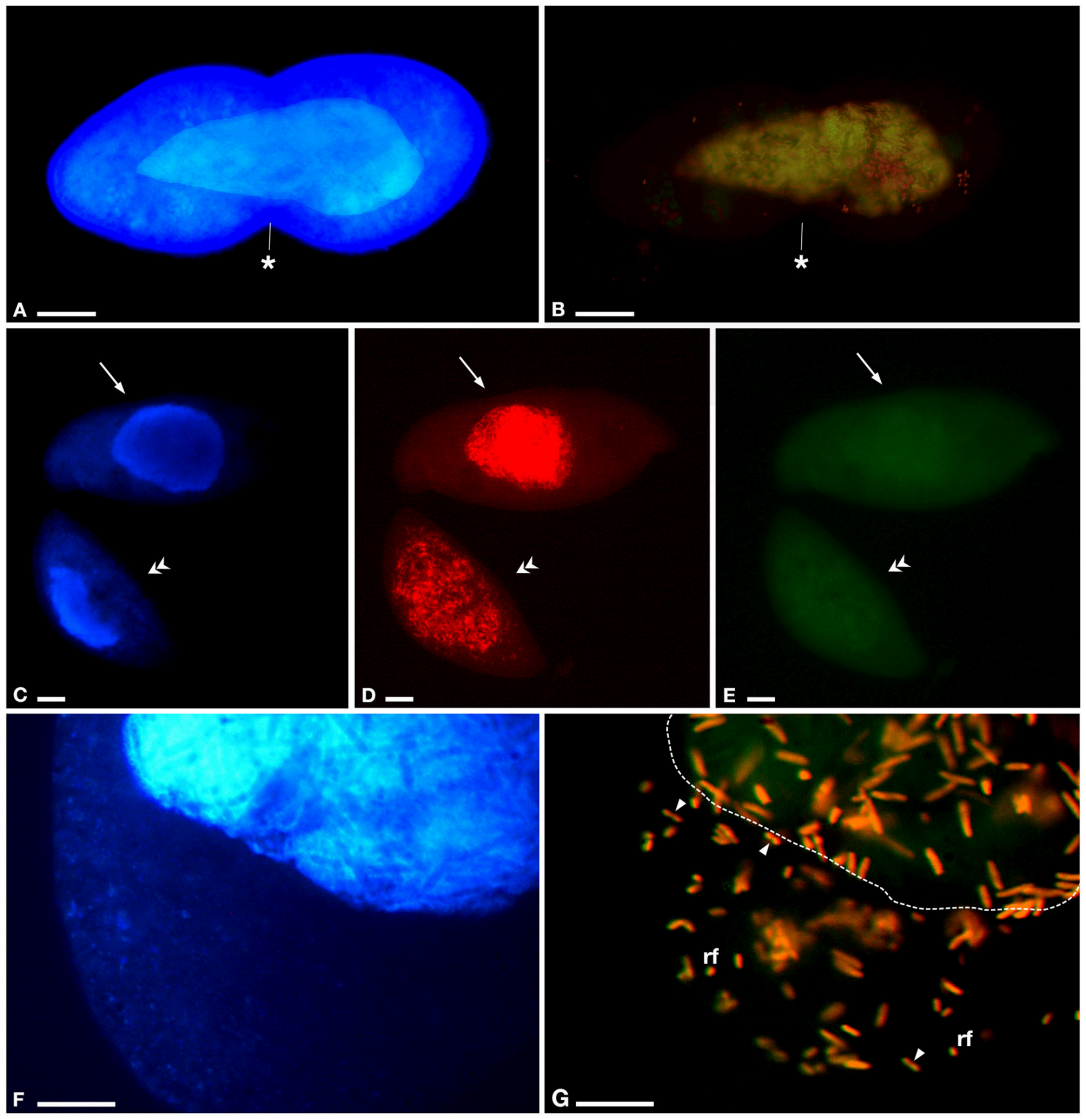
**Results of fluorescence *in situ* hybridization (FISH) on *P. multimicronucleatum* TP2**. Experiments were performed with different probe combinations: **(A,B)** GortProb659 (“*Ca*. Gortzia” genus-specific) and EUB338 (*Bacteria*), **(C-E)** GortzSha_1266 (“*Ca*. Gortzia shahrazadis” species-specific) and GortzInf_1268 (“*Ca*. Gortzia infectiva” species-specific), **(F,G)** GortzSha_1266 and EUB338. **(A)** Cell in division with infected macronucleus (*ma*), stained with 4,6-diamidino-2-phenylindole (DAPI), **(B)** positive to GortProb659 and EUB338 probes, completely overlapping. **(C)** Two cells fixed after 1year of culturing, showing intact *ma* stained with DAPI, **(D)** positive to GortzSha_1266, in *ma* (upper cell) and also in cytoplasm (lower cell), **(E)** same cells are negative to GortzInf_1268. **(F)** Closer view of cytoplasm and part of *ma* stained with DAPI of a *P. multimicronucleatum* cell infected by bacteria, **(G)** which are positive to GortzSha_1266 and EUB338 probes, with completely overlapped signals: reproductive forms (*rf*) and infectious forms well visible in host cytoplasm. *Arrows* indicate host cell with infection restricted to *ma* only, *double arrowheads* host cell with infection both in *ma* and in cytoplasm; *arrowheads* endosymbiont reproductive forms in cytoplasm during binary fission; *dotted line* position of *ma* inside host cell. The *asterisks* indicate the cleavage furrow. *Bars* stand for 20 μm **(A–E)** and 10 μm **(F,G)**.

Quite surprisingly, we found these HLB not only in the MA but also evenly distributed in the cytoplasm, inside and outside food vacuoles (Figures 1H,I, 4G, 5D,F,G); using TEM and FISH techniques we detected large numbers of IFs and TFs, but also some RFs (Figures 4G, 5G). This phenomenon was observed during a random check 4 months after sampling and occurred in 10–20% of analyzed cells (*n* = 40). Due to the low number of cells in our culture, it was not possible to periodically check the status of infection and its progress, however, 1 year after sampling this unusual cytoplasmic distribution was present in 50% of the analyzed cells (*n* = 42).

During host cell division, we never observed the “connecting piece” (Figures 1C,D, 5A,B). Endosymbionts were spread among daughter cells within MA pieces which, in many cases, were not equally shared in terms of size, suggesting that the bacteria interfere in host cell division.

During our cross-infection assay, we observed the presence of HLB inside the MA of *P. multimicronucleatum* PC6 48 h after the beginning of the experiment, showing the initial RF morphology observed in TP2 cells after a few weeks in cultivation (Figure 1L). From that moment, HLB cells in PC6 strain started to grow and differentiate, but after some days host cells started to decline and die.

Ultrastructural analysis of TP2 *P. multimicronucleatum* after 1 year of cultivation (Figure 4) supported live investigations using DIC microscope during the same period (Figures 1F–K). We were able to recognize different ultrastructural features characterizing the life-cycle stages described above: RF, IF, and TF. RF appeared short and roundish with the typical, homogeneous and relatively electron-transparent prokaryotic cytoplasm. The IF, longer and rod-shaped with slightly tapered ends, displayed differentiated cytoplasmic and periplasmic parts and a recognition tip-like structure in the apical part of the body (Figures 4A–C). In some cases, recognition tip-like structures were present at both ends of the cell (Figure 4A). The periplasmic regions of IF exhibited a very dense, osmiophilic pattern, while the recognition tip contained less osmiophilic material. The periplasm constituted a rather thin layer, often unevenly distributed beneath the outer cellular membrane of IF, sometimes forming outgrowths or sublayer invaginations in the cytoplasm (Figure 4F). We could discriminate at least two different periplasmic regions on the bases of their density: a darker, electron-dense part and a lighter one (Figures 4B,C). The IF cytoplasm was more heterogeneous and denser than the RF cytoplasm. Extensions of cytoplasm could be seen protruding into the periplasm in some cases (Figures 4B,C,F). Sometimes the same cell would manifest two or more stripes of dense periplasm, interposed with other cytoplasmic regions (probably forming the striped/dotted pattern previously observed via DIC microscopy). Another difference between IF and RF was their membrane composition: in the majority of IF the surface membrane was surrounded by fine fibrous material and, in some cases, manifests an additional membrane covering this fibrous-like layer (Figures 4C,F). TF appeared rod-shaped, with a size comparable to IF. The TF ultrastructure showed the presence of prokaryotic cytoplasm, without a distinctive periplasmic part (Figure 4E). The density of the TF cytoplasm resembles that observed in IF. Some TF manifest recognition tips at one or both ends of the body, as we observed in IF (Figure 4A). All forms showed a constant diameter of 0.7–0.8 μm.

**Figure 6:**
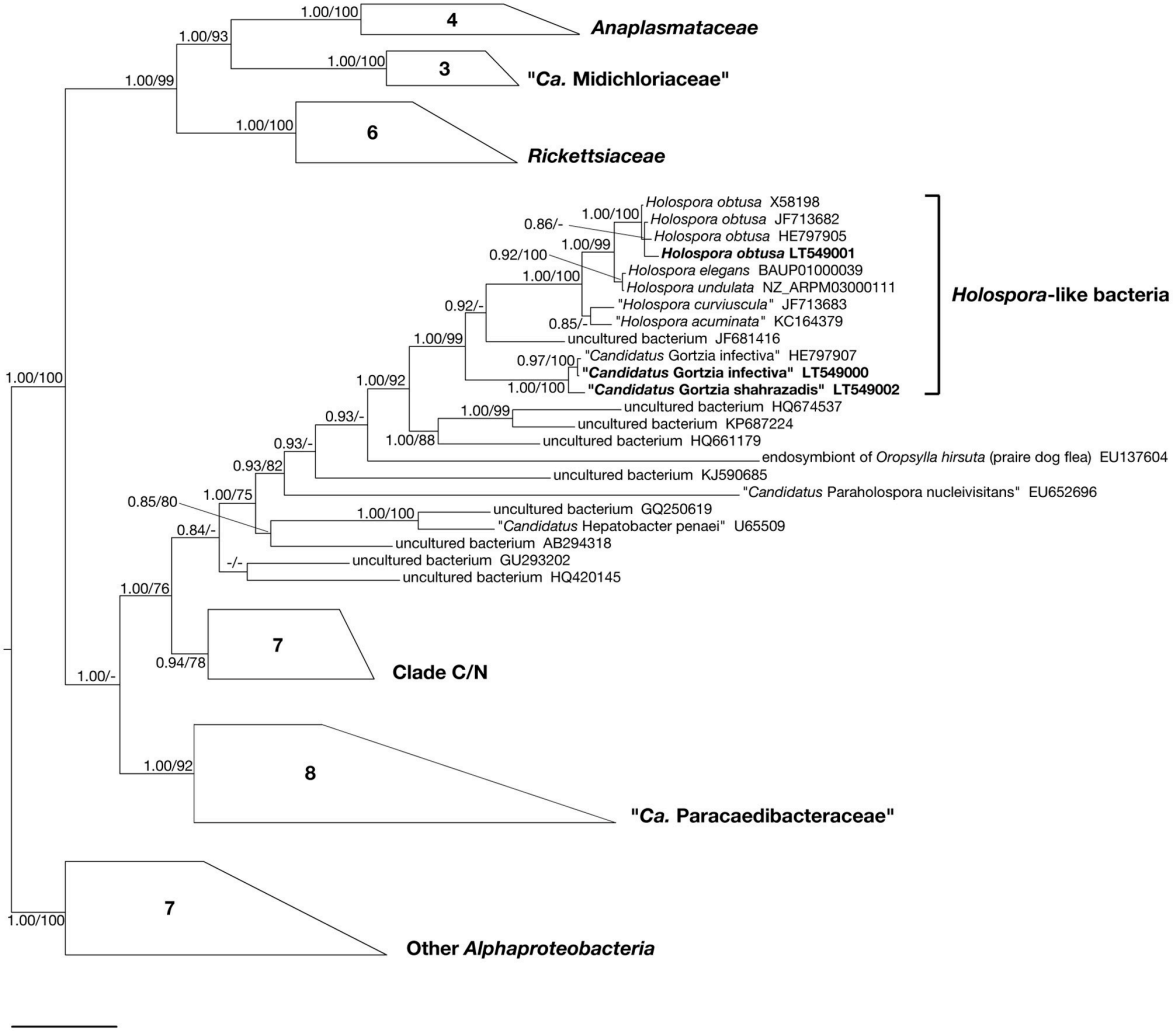
**Bayesian inference tree of the order *Rickettsiales* based on 16S rDNA sequences**. Numbers associated to nodes represent posterior probabilities and maximum likelihood bootstrap values, respectively (only values above 0.80–70 are shown). Numbers inside trapezoids correspond to sequences used to represent that clade. Sequences obtained in the present work are in *bold* characters. *Bar* stands for an estimated sequence divergence of 6%.

A 1398 bp long 16S rDNA sequence was obtained from this *P. multimicronucleatum* HLB and is available from ENA database under the accession number LT549002. From similarity matrix calculation we observed an identity value of 98.93% (15 mismatches) with “*Ca*. Gortzia infectiva” HE797907 and 90–91% with *Holospora* sequences (Table 2). In phylogenetic trees, this HLB species appeared strongly supported by statistical values as a member of the “*Ca*. Gortzia” clade (100/1.00), being a sister species of “*Ca*. Gortzia infectiva” (Figure 6).

**Table 2:** **Identity values among *Holospora*-like bacteria 16S rDNA sequences**.

	**a**.	**b**.	**c**.	**d**.	**e**.	**f**.	**g**.	**h**.	**i**.	**j**.	**k**.	**l**.
a. *H. obtusa*	_											
HE797905												
**b**. ***H. obtusa***	98.78	_										
**LT549001**												
c. *H. obtusa*	99.53	99.14	_									
JF713682												
d. *H. obtusa*	99.24	99.16	99.86	_								
X58198												
e. *H. elegans*	97.64	97.84	98.22	98.44	_							
BAUP01000039												
f. *H. undulata*	97.71	97.91	98.29	98.53	99.93	_						
NZ_ARPM03000111												
g. “*H. acuminata”*	95.88	95.26	96.39	96.05	96.27	96.34	_					
KC164379												
h. “*H. curviuscula”*	96.15	95.88	96.42	96.60	97.09	97.10	97.90	_				
JF713683												
i. Unc. Bact.	91.97	91.31	90.90	91.07	91.23	91.15	91.38	91.46	_			
JF681416												
j. “*Ca*. G. infectiva”	91.06	90.47	90.32	90.47	89.97	90.04	90.30	90.59	91.78	_		
HE797907												
**k. “*****Ca***. **G. infectiva”**	90.98	90.76	90.39	90.77	90.28	90.35	90.37	90.67	91.86	99.93	_	
**LT549000**												
**l. “Ca. G. shahrazadis”**	91.06	90.54	90.32	90.40	90.11	90.19	90.09	90.45	91.62	98.93	99.00	_
**LT549002**												

*Identity values obtained via distance matrix calculation by ARB program; sequences obtained in present work are shown in bold*.

Preliminary FISH analyses suggested the presence of members of the “*Ca*. Gortzia” genus in *P. multimicronucleatum* TP2 (Figures 5A,B). In a double hybridization experiment with the newly designed species-specific probes GortzSha_1266 and GortzInf_1268, HLB from *P. multimicronucleatum* showed a positive signal only to GortzSha_1266 and not to the specific probe for “*Ca*. Gortzia infectiva” (Figures 5C–E). Moreover, FISH results were positive to GortzSha_1266 and EUB338 probes, with completely overlapping signals (Figures 5F,G), suggesting that the new HLB was the only bacterial species harbored by the TP2 cells. As detected from live observations, in many cases we found bacteria positive to the GortzSha_1266 probe not only in the MA but also in the cytoplasm (Figure 5G), with a homogeneous distribution. No broken MA were detected, which could have explained this unusual distribution.

These results confirmed both the presence of HLB corresponding to our 16S sequences inside the *P. multimicronucleatum* host and that it is possible to discriminate this bacterium from the already characterized “*Ca*. Gortzia infectiva”, using species-specific probes. The sequence of probe GortzSha_1266 matches no sequences in the RDP database, while GortzInf_1268 matches 4 sequences belonging to “*Ca*. Gortzia infectiva” clones. Sequences of these two new probes were deposited into probeBase database (Greuter et al., 2016).

### “*Ca*. Gortzia infectiva” from India, endosymbiont of *P. jenningsi* BJ1

*Paramecium jenningsi* BJ1 showed a “*Ca*. Gortzia infectiva”-related infection in the MA, from images taken of live specimens and from FISH results (Figure 2). The 16S rDNA sequence from this endosymbiont, 1432 bp long, showed 99.93% of identity (1 mismatch) with “*Ca*. Gortzia infectiva” HE797907 from Thailand, with which it forms a monophyletic species-level group, supported by strong statistical values (100/0.97) (Figure 6). The identity values with the other two newly characterized endosymbiont sequences, TP2 and SH2, were 99.00 and 90.76%, respectively (14 mismatches and 115 mismatches plus 18 indels, respectively) (Table 2). Our sequence is available from ENA database under the following accession number: LT549000, “*Ca*. Gortzia infectiva” BJ1.

### *Holospora obtusa* from India, endosymbiont of *P. caudatum* SH2

Live observations, supported by molecular analysis, confirmed the presence of *H. obtusa* in the MA of *P. caudatum* SH2 (Figure 3), from which we obtained a 16S rDNA sequence of 1434 bp, available on ENA database under the accession number LT549001, *H. obtusa* SH2. The identity was 98.78–99.16% with other *H. obtusa* sequences (HE797905, JF713682, X58198), (12 mismatches - 11 mismatches/2 indels) (Table 2). Although this *H. obtusa* from India showed a slightly higher divergence in comparison with its previously known conspecifics, from the phylogenetic analysis these four sequences clearly form a monophyletic clade (Figure 6), supported by high values of bootstrap and posterior probability (100/1.00).

*Holospora obtusa* was the sister group of the *H. undulata* (NZ_ARPM03000111) and *H. elegans* (BAUP01000039) clade. Together with “*H. curviuscula*” (JF713683) and “*H. acuminata*” (KC164379) and one uncultured bacterium (JF681416) they formed a monophyletic lineage, sister clade of the “*Ca*. Gortzia” genus. The sequences of *H. undulata* and *H. elegans* showed 99.93% identity, differing by only 1 bp.

## Discussion

### Novel HLB from *P. multimicronucleatum* TP2

In the present study, we describe a novel HLB, belonging to the “*Ca*. Gortzia” genus. It occurred in *P. multimicronucletum*, a *Paramecium* species in which HLB have never been documented (Fokin and Görtz, 2009). This endosymbiont shares many traits with the *Holospora* and “*Ca*. Gortzia” genera, such as the peculiar life-cycle involving two different stages (IF, RF), and ultrastructural internal compartmentalization of IF (Görtz and Wiemann, 1989; Wiemann and Görtz, 1989). Furthermore, it can be included in the group of “*Ca*. Gortzia infectiva”, *H. caryophila*, “*H. bacillata*”, and “*H. curvata*”, due to the absence of a “connecting piece” and the presence of several membrane layers surrounding IF. This particular feature, never observed in those *Holospora* species able to induce the “connecting piece”, is probably related to host-invasion or a releasing mechanism (Fokin et al., 1996; Fokin and Sabaneyeva, 1997; Fokin, 2015). Moreover, as observed in “*H. bacillata*” (Fokin, 1989), IF were able to divide by binary fission. On the other hand, we detected ultrastructural differences with other HLB (Schmidt et al., 1987; Görtz et al., 1990; Wiemann and Görtz, 1991): IF of the new bacterium showed a darker, less homogeneous cytoplasm, an unevenly distributed periplasm with two distinguishable regions, and a recognition tip that did not show the additional subdivisions detected in “*Ca*. Gortzia infectiva” (Boscaro et al., 2013a). The presence of two recognition tip-like structures in IF and TF has been interpreted as an early stage of division for IF (since IF can divide) or differentiation and division, in the case of TF.

Our analyses emphasizes additional and surprising features, such as the simultaneous and extensive presence of bacteria both in the host cytoplasm and MA: this pattern has never been reported for any species of *Holospora* nor for “*Ca*. Gortzia infectiva” (Fokin and Görtz, 2009; Boscaro et al., 2013a). The presence of IF in the cytoplasm has previously been reported and interpreted as a secondary infection of the host or as IF trafficking through the cytosol to exit the cell (Fokin and Sabaneyeva, 1997; Fokin, 2015). The presence of dividing RF in the cytoplasm (Figure 5G) suggest that in this case, unique in HLB, the symbiont is able to complete its life-cycle in the cytoplasm. Further investigation is needed to resolve this issue.

The only organism with a similar behavior among phylogenetically relatives is “*Ca*. Paraholospora nucleivisitans”, a cytoplasmatic endosymbiont of *P. sexaurelia*, but observed to enter the MA of its host (Eschbach et al., 2009); but it was rarely present in both cell compartments simultaneous. Furthermore, it showed different morphological features and low 16S rDNA identity (83.95%) with all HLB, indicating that this feature is not a shared derived character of the two organisms.

The other new characteristic of this novel HLB, as mentioned above, is the highly variable size and shape of IF and TF, observed after several months of culturing. IF, indeed, showed an abnormal pattern of growth (it almost doubled its length in some cases) and bacterial periplasmic distribution, when compared to the classical HLB morphology. We do not yet know the cause for these unusual variations in IF/TF shape. The overall supposition is that “aberrant” IFs faced some physiological problem in developing the classical morphology. Indeed, the increased number of TFs in the MA could be caused by an inability to exit this stage, with a consequent increase in size. When they were able to develop cellular differentiation, the periplasmic part has been produced in an unevenly pattern. In other words, these traits could be due to physiological stress, disease, or mutations that accumulate in vertically inherited bacteria, with a probable reduction in the efficiency of horizontal transfer, or due to changes in host/symbiont interactions occurred under laboratory growth conditions. The fact that the classical HLB morphology is observed in the experimentally infected PC6 strain could be due either to a positive selection for non-altered bacteria, still able to perform horizontal infection correctly, or to the capability of bacteria to restore the initial cell morphology in a different, unaltered, host environment. Although these are just hypothesis, the observed features can be used for taxonomic purposes. From a molecular point of view, this new HLB is phylogenetically close to “*Ca*. Gortzia infectiva” from *P. jenningsi*. Our results suggest that “*Ca*. Gortzia infectiva” and the new HLB coexist in the same geographical region and may have host specificity, a feature common in the sister genus *Holospora*.

To conclude, for this HLB we suggest the status of a novel species for the following reasons: 1) the different morphology and the striking phenotypic plasticity, never observed before in HLB; 2) the ability to infect both the MA and the cytoplasm; 3) the new host species in which it has been found, *P. multimicronucleatum*, given the fact that species-specificity between host and endosymbiont is well-known for most HLB; 4) the 16S rDNA distance from closely related species, which is compatible with threshold values proposed for new species description (Rosselló-Móra and Amann, 2015). For this new HLB species, belonging to the “*Ca*. Gortzia” genus, we propose the name “*Candidatus* Gortzia shahrazadis”, accordingly with Murray and colleagues (Murray and Schleifer, 1994; Murray and Stackebrandt, 1995). This specific name was chosen due to the Asian origin of this species and because one of the authors is known to say “I will finish this paper after one thousand and one nights.” A description of this new species is present at the end of the discussion.

### “*Ca*. Gortzia infectiva” and *Holospora obtusa* from India

We detected HLB in an Indian *Paramecium* population. A first analysis of phenotypic characters and host specificity led to the species attributions “*Ca*. Gortzia infectiva” and *H. obtusa*, respectively, from *P. jenningsi* and *P. caudatum*. In both cases, the typical IF and RF were observed inside the host MA, with typical size and features described in the literature (Görtz et al., 1989; Boscaro et al., 2013a). Molecular analyses was performed on fixed material and, in both cases, results were congruent with live observations, confirming the species assignment, despite both *Paramecium caudatum* 18S rDNA sequence and its *H. obusa* 16S rDNA sequences showed some peculiarities when compared to sequences of conspecific organisms, suggesting a level of divergence. Phylogenetic analysis showed clear association of the Indian *H. obtusa* with its conspecifics sampled in other countries. Nevertheless, according to branch lengths, Indian *H. obtusa* SH2 showed a clear divergence from northern strains.

In our trees all HLB cluster together: *Holospora* clade appeared as sister group of the “*Ca*. Gortzia” genus. In general, our phylogeny of the order *Rickettsiales* is in agreement with previously published analyses (Boscaro et al., 2013a; Hess et al., 2016). Additionally, we note that the sequences belonging to *H. undulata* and *H. elegans* are very similar. While past analyses included a short partial sequence (479 bp) from *H. elegans* (AB297813) (e.g., Boscaro et al., 2013a), to our knowledge this is the first study reporting the full-length gene sequence of this species, derived from its genomic assembly (Dohra et al., 2014). On the basis of the present data and given the fact that both *H. undulata* and *H. elegans* have been found in MI of *P. caudatum, H. undulata*—which has an unusual undulated shape in comparison to other *Holospora*—could be a morphotype of the species *H. elegans*. Nevertheless, further investigation is needed, including analysis of a greater number of strains and comparison at the whole-genome level. To our knowledge, the several strains of *H. undulata* molecularly characterized by our and other laboratories are all identical to the presently published 16S rDNA sequences (data not shown), while for *H. elegans* only a single complete sequence is available (Dohra et al., 2014). In any case, since these two species were described in 1890 by Hafkine (Hafkine, 1890), it seems particularly important to clarify this issue.

### Reconsideration about HLBs distribution

Our data confirm the presence of HLB endosymbionts in *Paramecium* species inhabiting different and distant locations in India. *P. multimicronucleatum* (TP2) was collected from freshwater Kolleru lake in Andhra Pradesh; *P. jenningsi* (BJ1) was sampled in Chilka Lake, a brackish lagoon in Odisha; and *P. caudatum* (SH2) was found in a freshwater stream at the top of Araku Hills (Andhra Pradesh). All these findings strongly suggest a relatively common presence of HLB in Indian ciliate populations, adapted to different types of tropical habitats.

**Figure 7:**
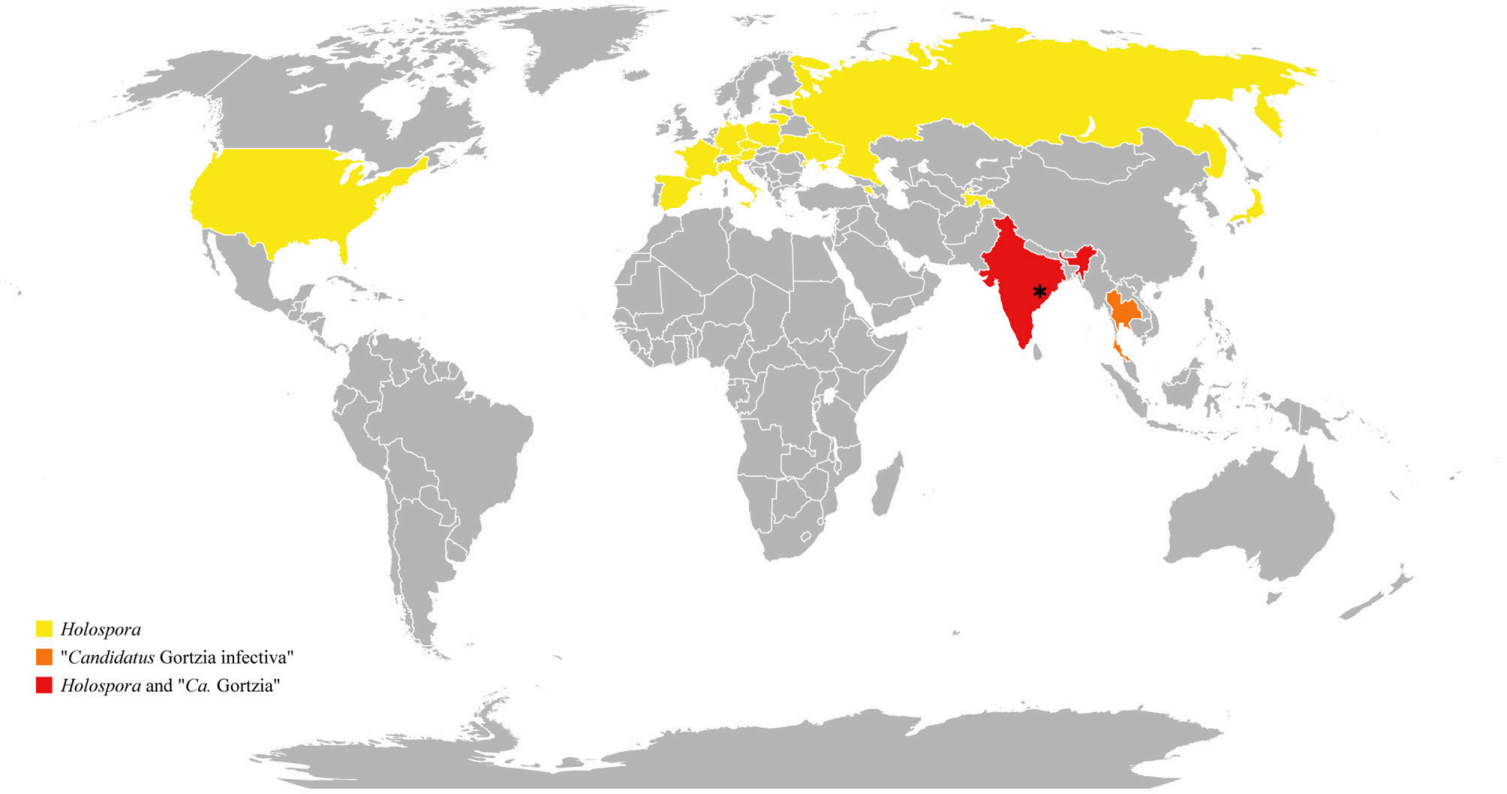
***Holospora*-like bacteria distribution**. *Yellow, orange*, and *red* indicate countries in which *Holospora*-like bacteria have so far been detected, whereas a *gray* indicates those areas not yet investigated for their presence. *Asterisk* indicates sampling area of *Holospora obtusa* and “*Ca*. Gortzia” species characterized in the present work.

To date *Holospora* has been considered an endosymbiont adapted to cold and temperate countries. It has been demonstrated that transmission efficiency of *H. undulata*, for example, is strongly affected by temperature, being more effective at 10°C than at 23 and 30°C (Fels and Kaltz, 2006). Moreover, *Holospora* was never found in tropical habitats. For all these reasons, our data introduce new and interesting information about HLB's distribution, being the lowest latitude record for the *Holospora* genus reported (Figure 7; Table 3).

**Table 3:** **Biodiversity and distribution of *Holospora*-like bacteria from ciliate hosts**.

**Name**	**Host**	**Localization**	**Distribution**	**References**
“*H. acuminata”*	*Paramecium bursaria*	MI	Armenia (Sevan Lake), Estonia, France (Paris), Germany (Munster, Simmelried), Russia (Kalininingrad, Karelia Republic, Pskov, Saint Petersburg, Uglich), Ukraine (Vinnytsia), USA	Ossipov et al., 1980; Kreutz and Foissner, 2006; Fokin and Görtz, 2009; Rautian and Wackerow-Kouzova, 2013; Lebedeva, personal communication
“*H. bacillata”*	*P. nephridiatum, P. calkinsi*	MA	Russia (Sredny Island-White Sea)	Fokin, 1989, 1993; Fokin et al., 1996; Fokin and Görtz, 2009
*H. caryophila*	*P. aurelia, P. caudatum*	MA	Austria (Innsbruck), Czech Republic (Karlovy Vary), Germany (Freiburg, Karlsruhe, Munster, Süderfahrenstedt), Italy (Milan), Russia (Baikal Lake, Irkutsk, Chuvashia, Kaliningrad, Pskov, S. Petersburg), Ukraine (Vinnytsia), USA (Boston)	Preer, 1969; Preer et al., 1974; Preer and Preer, 1982; Görtz, 1987; Fokin, 1993; Fokin and Görtz, 2009; Schrallhammer et al., 2013; Fokin, Lebedeva, personal communication
“*H. curvata”*	*P. calkinsi*	MA	Russia (Rjazkov Island-White Sea)	Fokin, 1993, 1997 Fokin and Görtz, 2009
“*H. curviuscula”*	*P. bursaria*	MA	Estonia (Tallinn), France, Germany (Stuttgart), Russia (Astrakhan Reserve, Karelia Republic, S. Petersburg, Seskar Island, White Sea)	Borchsenius et al., 1983; Skoblo and Lebedeva, 1986; Fokin et al., 1996; Fokin and Görtz, 2009; Vakkerov-Kouzova and Rautian, 2011; Lebedeva, personal communication
*H. elegans*	*P. caudatum*	MI	France (Paris), Germany (Munster), Italy (Sicily), Russia (Moshchny Island)	Hafkine, 1890; Görtz and Diekmann, 1980; Preer and Preer, 1982; Fokin and Görtz, 2009; Lebedeva, personal communication
*H. obtusa*	*P. caudatum*	MA	Estonia (Saarema Island), France (Paris), Germany (Bensersiel, Munster), **India (Araku Hills)**, Japan (Yamaguchi), Lithuania (Vilnius), Poland, Russia (Belgorod, Chernyakhovsk, Kaliningrad, Morskoje, Ropsha, S. Petersburg, Sinyavino, White Sea, Irkutsk, Sosnovy Bor, Vladivostok), Tajikistan, Ukraine (south part of Dnepr river), USA	Hafkine, 1890; Fiveiskaja, 1928; Gromov and Ossipov, 1981; Fokin et al., 1996; Fokin and Görtz, 2009; Vakkerov-Kouzova and Rautian, 2011; Lebedeva, personal communication; **present work**
“*H. recta”*	*P. caudatum*	MI	Russia (S. Petersburg)	Fokin, 1991; Fokin and Görtz, 2009
*H. undulata*	*P. caudatum*	MI	Estonia (Saarema Island), France (Paris), Germany (Munster, Stuttgart), Lithuania (Vilnius), Poland (Krakow), Russia (Kaliningrad, Morskoje, Moshchny Island, Nachodka, Ropsha, S. Petersburg, Sinyavino), Spain (Madrid), Ukraine (south part of Dnepr river), USA (Boston)	Hafkine, 1890; Gromov and Ossipov, 1981; Fokin et al., 1996; Fokin and Görtz, 2009; Lebedeva, personal communication
*Holospora* sp. 1	*P. putrinum*	MA	Germany (Karlsruhe); Russia (Yakutia Republic)	Fokin et al., 1996, 1999; Fokin and Görtz, 2009; Rautian et al., 2015
*Holospora* sp. 2, sp. 3	*Frontonia leucas, F. salmastra*	MA	Italy (Pisa, Serchio river)	Fokin et al., 2006; Ferrantini et al., 2007; Fokin and Görtz, 2009
“*Candidatus* Gortzia infectiva”	*P. jenningsi*	MA	Thailand (Cheweng Lake), **India (Chilka Lake)**	Boscaro et al., 2013a; **present work**
“*Candidatus* Gortzia shahrazadis”	***P. multimicronucleatum***	MA	**India (Kolleru lake)**	**Present work**

*New elements about HLB ecology and distribution from present work are shown in bold; MA Macronucleus, MI Micronucleus*.

Nevertheless, the stream from which we sampled *H. obtusa* was located on the top of Araku hills, 700 m above the sea level, where climatic conditions were more temperate than those in the lowlands. We hypothesize that *Holospora* species are found in tropical countries, but in areas with more temperate environments, such as hills or mountains, generating a patchy distribution. The relatively high 16S rDNA divergence of this specific *Holospora* population could result from a small population size and possible genetic isolation due to patchy and unconnected distributions.

In contrast, members of “*Ca*. Gortzia” clade have been found exclusively in tropical areas at sea level (Boscaro et al., 2013a). These results lead to the conclusion that the “*Ca*. Gortzia” genus is more adapted to tropical climates than *Holospora*.

From an ecological perspective, our results address the possible function of HLB in *Paramecium* eco-physiology. Some have identified *Holospora* as true parasites (Görtz, 1988; Lohse et al., 2006), while others emphasize that those bacteria confer resistance to the host cell against challenging environmental conditions, such as salinity (Smurov and Fokin, 1998) or temperature variations (Hori and Fujishima, 2003). *H. obtusa* is able to enhance heat-shock gene expression of the host *P. caudatum* when exposed to increasing temperatures during laboratory experiments (Hori and Fujishima, 2003). Thus, it is possible that HLB confer a degree of benefit to their host if exposed to temperature stress, although this hypothesis is controversial (Duncan et al., 2010).

Obviously, another necessary consideration is that the biogeographic distribution of HLB is highly dependent on their hosts' distribution. The *Paramecium* genus is cosmopolitan, although some species seem to be adapted to certain climatic conditions. *P. jenningsi*, for example, has been found mostly in tropical countries, whereas *P. caudatum* is reported mainly from northern areas with temperate climates (Wichterman, 1986; Fokin, 2010/11). However, fewer sampling efforts in tropical countries strongly affects the present understanding of ciliate distribution, and thus the distribution of their endosymbionts (Görtz, 2008; Fokin and Sera, 2014).

To conclude, our record of *H. obtusa* from India reshapes the known *Holospora* distribution and underlines the need for further sampling and research efforts, to better clarify the ecological significance of HLB.

### Emended description of “*Candidatus* Gortzia” genus (Boscaro et al., 2013a)

*Gortzia* (Gor'tzi.a; N.L. fem. n. *Gortzia*, in honor of Professor Emeritus Hans-Dieter Görtz). Gram-negative, *Alphaproteobacteria, Rickettsiales*, belonging to *Holospora*-like bacteria clade, together with *Holospora* genus and some other uncultured organisms. Macronuclear endosymbiont of *Paramecium* spp. (*P. jenningsi, P. quadecaurelia, P. multimicronucleatum*), and also cytoplasmatic in case of *P. multimicronucleatum*. “*Ca*. Gortzia”, has two different stages in its life-cycle: the short reproductive form (RF) and the elongated, rod-shaped infectious form (IF). IF showed cellular subcompartments: cytoplasm, periplasm, recognition tip. Periplasm and recognition tip could manifest parts with different electron-densities, according with species. No “connecting piece” or killer traits detected. The type species is “*Ca*. Gortzia infectiva” (Boscaro et al., 2013a). Only another species has been described: “*Ca*. Gortzia shahrazadis” (present work). Basis of assignment: positive matching with the 16S rRNA-targeting oligonucleotide genus-specific probe GortProb659 (5′-TTCCGTTTTCCTCTACCA-3′).

### Description of “*Candidatus* Gortzia shahrazadis”

*Gortzia shahrazadis* (Gor'tzi.a shah.ra.za'dis; N.L. fem. n. *Gortzia*, in honor of Professor Emeritus Hans-Dieter Görtz; N.L. adj. *shahrazadis*, of Shahrazad, main character and charming story teller in the Arabian collection of tales, “The One Thousand and One Nights”). Mainly macronuclear endosymbiont which can sometimes be observed in the cytoplasm of *P. multimicronucleatum*, sampled from Kolleru, a freshwater lake in India. It has two life-cycle stages: a small, reproductive form (RF: 2.5–3.3 μm) and a rod-shaped infectious form (IF). The latter shows cellular compartmentalization: cytoplasm, periplasm (with two areas distinguishable by different density), and recognition tip. Two morphotypes of IF are detected during different periods of laboratory culturing: a shorter IF at the beginning (6.9–10.7 μm), and a longer one (8.2–14.7 μm) with irregular distribution of periplasm after 1 year of cultivation. The latter are found together with a high number of transient forms (TF), which are long (6.7–15.5 μm), rod-shaped and not yet differentiated into IF at an ultrastructural level. All forms showed a constant diameter of 0.7–0.8 μm. No production of a “connecting piece” during host cell division was observed. Several membranaceous layers surrounded fully differentiated IF. Basis of assignment: 16S rRNA gene sequence (ENA database, accession number LT549002) and positive matching with the 16S rRNA-targeting oligonucleotide probe GortzSha_1266 (5′-TTTTGATTTGCTCAAGGTCGC-3′).

## Author contributions

VS, SF, CB, and VN actively performed sampling in India. CB, VN, BS, CK organized and logistically supported sampling activity in India, VS carried out most of FISH experiments, molecular and phylogenetic analyses, probes and primers design. SF performed preliminary microscopic observation finding all the endosymbionts, morphologically identified the hosts, and performed TEM analysis. MC helped to realize the phylogenetic study. CB and VN performed part of the molecular characterization. SF, FV, BS, CK, and GP carefully supervised all the experiments and gave suggestions for data interpretation. VS wrote the manuscript. All the authors were involved in manuscript revision. GP coordinated the whole research activity.

## Funding

This work was supported by the European Commission FP7-PEOPLE-2009-IRSES project CINAR PATHOBACTER (247658) and by the PRIN fellowship (protocol 2012A4F828_002) from the Italian Research Ministry (MIUR), and by the European Commission FP7 post grant Open Access Pilot Open AIRE.

### Conflict of interest statement

The authors declare that the research was conducted in the absence of any commercial or financial relationships that could be construed as a potential conflict of interest.
