# Supplementary material for: “Candidatus Gortzia shahrazadis”, a Novel Endosymbiont of Paramecium multimicronucleatum and a Revision of the Biogeographical Distribution of Holospora-Like Bacteria
Source: Front Microbiol. 2016 Nov 4;7:1704. doi: 10.3389/fmicb.2016.01704 (PMC5095128; doi:10.3389/fmicb.2016.01704)
Supplement: Supplementary file 1 [file Table1.DOCX]

**Supplementary material**

|  | | |
| --- | --- | --- |
| **Table S1 \| List of 16S rDNA sequences used for phylogenetic analysis, not shown in tree figure** | | |
| **Clade** | **Species** | **Accession number** |
| *Anaplasmataceae* | *Anaplasma ovis* | AF318945 |
|  | “*Candidatus* Neoehrlichia mikurensis” | AB196305 |
|  | *Ehrlichia ruminantium* | NR074513 |
|  | *Wolbachia* endosymbiont of *Drosophila melanogaster* | AE017196 |
|  |  |  |
| “*Candidatus* Midichloriaceae” | “*Candidatus* Midichloria mitochondrii” | AJ566640 |
|  | Endosymbiont of *Acanthamoeba* sp. | AF069963 |
|  | “*Candidatus* Lariskella arthropodarum” | AB624350 |
|  |  |  |
| *Rickettsiaceae* | *Rickettsia canadensis* | NR074485 |
|  | *Rickettsia rickettsii* | M21293 |
|  | “*Candidatus* Trichorickettsia mobilis” | HG315612 |
|  | “*Candidatus* Megaira polyxenophila” | AJ630204 |
|  | *Orientia tsutsugamushi* | D38623 |
|  | “*Candidatus* Cryptoprodotis polytropus” | FM201295 |
|  |  |  |
| Clade C/N | *Caedibacter caryophilus* | AY753195 |
|  | *Caedibacter macronucleorum* | AM236091 |
|  | “*Candidatus* Caedibacter acanthamoebae” | CP008936 |
|  | Uncultured bacterium | EU803767 |
|  | “*Candidatus* Nucleicultrix amoebiphila” | KF697195 |
|  | Uncultured bacterium | HQ697417 |
|  | Uncultured bacterium | FJ529991 |
|  |  |  |
| “*Candidatus* Paracaedibacteraceae” | “*Candidatus* Paracaedibacter symbiosus” | AF132139 |
|  | Uncultured bacterium | FJ517710 |
|  | “*Candidatus* Paracaedibacter acanthamoebae” | AF132137 |
|  | “*Candidatus* Odyssella thessalonicensis” | AF069496 |
|  | Uncultured bacterium | AJ318202 |
|  | “*Candidatus* Finniella lucida” | KT343635 |
|  | “*Candidatus* Finniella inopinata” | KT343636 |
|  | “*Candidatus* Captivus acidiprotistae” | AF533506 |
|  |  |  |
|  | | |
